# Time-Frequency, Complexity, and Fractal Analyses of Hemoglobin and Deoxyhemoglobin Responses to Quantify Mechanisms of Actions of Cupping Therapy

**DOI:** 10.3390/e28060597

**Published:** 2026-05-27

**Authors:** Nasrin Dabirian, Mansoureh Samadi, Amir Babaniamansour, Yameng Li, Manuel E. Hernandez, Yih-Kuen Jan

**Affiliations:** 1 Department of Health and Kinesiology, University of Illinois at Urbana-Champaign, Urbana, IL 61801, USA; 2 Department of Physical Education, Northwestern Polytechnical University, Xi’an 710072, China; 3 Department of Biomedical and Translational Sciences, Carle Illinois College of Medicine, University of Illinois at Urbana-Champaign, Urbana, IL 61801, USA

**Keywords:** complexity, entropy, near infrared spectroscopy, negative pressure, wavelet

## Abstract

Cupping therapy has been demonstrated to improve hemodynamic regulation. Existing studies have reported mean changes of oxyhemoglobin (OxyHb) and deoxyhemoglobin (DeoxyHb), which do not capture the multi-scale regulatory dynamics of the microvasculature. It is therefore unclear whether cupping therapy modulates the complexity and fractal property of hemodynamic signals. The objective of this study was to examine complexity of hemodynamic response to cupping therapy. A 2 by 2 factorial design with repeated measures was used to examine the main effect of pressure (−225 and −300 mmHg) and duration (5 and 10 min) and their interaction. A near infrared spectroscopy (NIRS) was used to measure OxyHb and DeoxyHb concentrations before and after cupping therapy. A total of 18 healthy participants were enrolled in this study. The wavelet analysis, sample entropy and detrended fluctuation analysis (DFA) were used to quantify the oscillatory, complexity, and fractal scaling properties of OxyHb and DeoxyHb signals. A two-way ANOVA with Bonferroni correction was used to examine the main and interaction effects. The results demonstrated that the combined effects of pressure and duration, rather than either factor independently, were the primary determinants of the dynamic hemodynamic response to cupping therapy, with significant Pressure × Duration interactions observed in DeoxyHb myogenic wavelet power (*F* = 4.636, *p* = 0.046, η^2^p = 0.214), OxyHb (*F* = 5.704, *p* = 0.029, η^2^p = 0.251) and DeoxyHb (*F* = 6.600, *p* = 0.020, η^2^p = 0.280) sample entropy, and DeoxyHb DFA scaling exponent (*F* = 5.598, *p* = 0.030, η^2^p = 0.248). In addition, cupping pressure selectively modulated neurogenic DeoxyHb oscillatory power (*F* = 5.001, *p* = 0.039, η^2^p = 0.227), and cupping duration significantly altered the fractal scaling properties of DeoxyHb signals (*F* = 7.775, *p* = 0.013, η^2^p = 0.314). The findings indicate that the interaction of pressure and duration of cupping therapy could effectively modulate hemodynamic responses. To the best of our knowledge, this is the first study investigating the complexity of hemodynamic responses after cupping therapy.

## 1. Introduction

Cupping therapy is one of the oldest documented manual therapies, with formal use in traditional Chinese, Unani, and Middle Eastern medical systems and a growing presence in modern complementary and integrative care [1,2]. In traditional Chinese medicine (TCM), cupping therapy is grounded in the principles of unblocking the meridians to restore neural and circulatory regulation and promoting blood circulation and removing blood stasis [1,3] regulatory processes that modern physiological signal analysis can now quantify directly at the microvascular level. Contemporary classifications group cupping techniques into “dry” (suction only) and “wet” (suction with skin scarification and bloodletting), with sub-types based on the suction-generation mechanism, fire/heat, manual hand-pump, and electronic vacuum pumps, and on whether cups are stationary, moving, or pulsatile [2]. Clinically, cupping has been applied to musculoskeletal and pain-related conditions, including chronic non-specific neck pain [4,5], chronic low back pain, and post-exercise recovery in amateur and professional athletes [6,7]. A meta-analysis of 18 trials (*n* = 1172) found cupping reduced pain (SMD = −1.03) and disability (SMD = −0.66) versus no treatment, but not versus sham [1]. In musculoskeletal and sports rehabilitation settings, cupping is typically used as an adjunct to exercise and manual therapy, with low-to-moderate evidence supporting reductions in pain and improvements in range of motion [6]. These reviews consistently identify inconsistent dosing of pressure and duration as a key limitation, highlighting the need for studies that quantify dose-response relationships.

The leading mechanistic hypotheses for cupping converge on the local effects of sustained negative pressure on the skin–fascia–muscle unit [8,9,10]. Finite-element modeling by Tham et al. demonstrated that cup suction produces large tensile and shear stresses that propagate through skin into subcutaneous fat and superficial muscle, with the magnitude scaling directly with vacuum pressure and cup geometry [8]. This mechanical loading is thought to stretch fascia, decompress trigger points, and rupture small superficial capillaries, producing the characteristic ecchymotic marks, while simultaneously triggering vasodilation, reactive hyperemia, and increased local microcirculatory perfusion of skin and muscle [9,10]. From a neurophysiological perspective, the strong cutaneous afferent input generated by suction has been proposed to modulate pain via gate-control and diffuse noxious inhibitory control (DNIC) pathways, raising mechanical pain thresholds in the treated dermatome [5,9]. Additional putative mechanisms include local upregulation of the heme oxygenase/biliverdin/carbon monoxide cytoprotective system, modulation of inflammatory and oxidative-stress markers, and immunomodulation through controlled microtrauma [9,10]. Although these candidate mechanisms are biologically plausible and partially supported by animal and human work, most remain inferred rather than directly measured; quantitative, dose-resolved physiological readouts are therefore needed to discriminate among them [11].

Near infrared spectroscopy (NIRS) uses the differential absorption of near-infrared light (700–900 nm) by oxyhemoglobin (OxyHb) and deoxyhemoglobin (DeoxyHb) to track microvascular concentration changes at 1–10 Hz, with continuous-wave systems sampling tissue at roughly half the source–detector separation, about 1–1.5 cm at a typical 2.5–3 cm spacing [12,13]. In the skeletal muscle, OxyHb is more strongly weighted toward arteriolar and capillary inflow and tissue oxygen delivery, whereas DeoxyHb reflects venular pooling and the balance between O_2_ extraction and washout, so concurrent tracking captures both supply and demand [14]. Early NIRS studies using embedded or adjacent sensors documented rapid cupping-induced hemodynamic changes, demonstrating that suction significantly alters local OxyHb and DeoxyHb concentrations [15,16]. More recent multi-channel, dose-controlled work has shown that cupping pressure, duration, and cup size each produce significant main and interaction effects on biceps OxyHb and DeoxyHb, and that hemodynamic coupling between cupped and adjacent tissue strengthens with higher pressure and longer duration [17]. Across this literature, however, the dependent measures have been almost exclusively mean concentration changes; the time–frequency structure and complexity of OxyHb/DeoxyHb signals during cupping remain unquantified.

Blood-flow and hemoglobin signals contain rich oscillatory and fractal structure that mean concentration changes alone cannot capture. The continuous wavelet transform, developed by Stefanovska and colleagues, decomposes peripheral microvascular oscillations into five physiologically distinct frequency bands, endothelial, neurogenic, myogenic, respiratory, and cardiac, and has been operationalized to quantify these regulatory contributions to skin blood flow, including after cupping therapy [18,19,20]. Complementing these spectral methods, sample entropy (SampEn) measures signal regularity [21], detrended fluctuation analysis (DFA) characterizes long-range temporal correlations via a scaling exponent [22], and multiscale entropy (MSE) extends complexity quantification across multiple timescales [23,24], together forming an established toolkit for cardiovascular and microvascular signals. Although these wavelet and other methods have been applied to NIRS signals in cerebral and muscular contexts, they have not been combined to interrogate cupping-induced OxyHb/DeoxyHb dynamics; the present study addresses this gap by applying an integrated time-frequency and complexity, and fractal scaling framework to quantify the dose-dependent hemodynamic response to cupping.

Taken together, cupping therapy produces measurable clinical and hemodynamic effects, yet three gaps remain: dose–response relationships between cupping pressure and duration are poorly resolved; prior NIRS work has relied almost exclusively on mean OxyHb and DeoxyHb concentration changes; and wavelet-based time-frequency and complexity methods, though validated for microvascular signals in other contexts, have not been applied to cupping-induced hemodynamics. The present study therefore asks the following questions: (1) How do cupping pressure and duration modulate the time-frequency structure of OxyHb and DeoxyHb signals within the endothelial, neurogenic, and myogenic bands? (2) How do these parameters affect signal complexity, as quantified by sample entropy and detrended fluctuation analysis? We hypothesized that higher pressures and longer durations would (i) increase wavelet amplitudes in the endothelial, neurogenic, and myogenic bands, and (ii) produce dose-dependent changes in sample entropy and DFA scaling exponents, with significant main and interaction effects of pressure and duration.

To the best of our knowledge, this is the first study to investigate the influences of cupping pressure and duration on oxyhemoglobin and deoxyhemoglobin responses using time-frequency analysis (wavelet transform), complexity analysis (sample entropy), and fractal scaling analysis (detrended fluctuation analysis). Time-frequency analysis enabled evaluation of the underlying physiological regulation (endothelial, neurogenic, and myogenic controls), while complexity and fractal scaling analyses quantified the dynamic structure and long-range organization of the oxyhemoglobin and deoxyhemoglobin responses to cupping therapy.

## 2. Material and Methods

A two-way factorial study design with crossover design was used in this study to investigate the interactions between cupping pressures and cupping durations on hemoglobin and deoxyhemoglobin responses at the area inside the cupping cup. This factorial design is consistent with the analytical framework used in previous studies from our group investigating the effects of cupping pressure and duration on muscle hemodynamic responses using near-infrared spectroscopy. Our previous studies [25,26] have reported the mean values of oxyhemoglobin and deoxyhemoglobin. In this study, we reported the hemodynamic kinetics from this project.

### 2.1. Participant

This human subject research was approved by the Institutional Review Board, University of Illinois at Urbana-Champaign (IRB #22900). All participants gave written consent before participating in this study. The sample size estimation was carried out using G*Power (version 3.1.9.7; Franz Faul, Universität Kiel, Kiel, Germany) under ANOVA: repeated measures, within factors for a priori power analysis using effect size 0.4, α 0.05, power 0.8, number of groups 1, and number of measurements 4 for a total of 10 participants. The choice of a large effect size of 0.4 was based on our previous study demonstrating that cupping therapy can effectively induce local hemodynamic responses. Inclusion criteria were healthy adults without any diagnosis of cardiovascular disease and aged from 18 to 40 years. Exclusion criteria were open wounds, scars, or tattoos over the tested area, diagnosis of diabetes, any neuromuscular impairments, or smokers.

### 2.2. Instrumentation

An automatic suction pump (P1000-PCS, California Medical Device Manufacturing Facility, Sunnyvale, CA, USA) was used to produce negative pressure inside the cup (inside diameter 45 mm and curved outer diameter as 53–55 mm). The negative pressure device can produce negative pressure ranging from 0 to −760 mmHg by automatic suction.

A functional NIRS (fNIR Imager 1000, fNIR Devices, Potomac, MD, USA) was used to measure oxyhemoglobin and deoxyhemoglobin concentrations (in µM). The sensor pad consisted of 10 photoreactors and 4 NIR LDF light sources for a total of 16 channels. The light source and detector distance is 2.5 cm, which allows about 1.25 cm measurement depth [25,26]. The fNIRS sensor pad was placed on the biceps brachii muscle. A predefined region about 4 channels was carefully aligned with the area covered by the cupping cup. The sampling frequency was 2 Hz. The raw fNIRS signals were low-pass filtered with a finite impulse filter of cut-off frequency at 0.14 Hz to eliminate possible respiration and heart rate signals [25,26]. The fNIRS values of 5 min pre-cupping period were averaged to calculate the relative changes of fNIRS signals in response to cupping therapy to overcome individual’s baseline variations.

### 2.3. Experimental Protocols

All experimental procedures were performed in the Rehabilitation Engineering Lab at the University of Illinois at Urbana-Champaign. The lab was maintained in a thermoneutral temperature (25 °C). A research participant visited the lab 4 times to complete this study, and each visit was separated by about 48 h. Before coming to the lab, a participant was instructed to avoid exercise and caffeine drinks. The sensor pad was placed on the biceps brachii of the dominant hand. The choice of the dominant hand was because the long-term goal of this research is to reduce muscle fatigue of the dominant hand after sports. During the experiment, a participant laid on a mat table and the arm extended and fully pronated. The baseline measure (pre-cupping period) was 5 min to establish the baseline concentration of hemoglobin and deoxyhemoglobin. The rationale to choose 10 min post-cupping therapy was based on existing studies demonstrating a fully recover of 10 min cupping therapy [25,26]. Then, one of 4 protocols (−225 mmHg for 5 min, −225 mmHg for 10 min, −300 mmHg for 5 min, and −300 mmHg for 10 min) was randomly assigned to a participant. Several procedures were used to decrease biases, including the participant being blinded for 4 cupping therapy protocols and that the researcher performing the data collection was not involved in the hypothesis development.

### 2.4. NIRS Signal Processing

#### 2.4.1. Wavelet Transform

Continuous wavelet transform (CWT) was applied to decompose the OxyHb and DeoxyHb time series into time-frequency representations. The CWT is defined as [19]

(1) $$w(s,t)=\int_{-\infty }^{\infty }\psi_{s,t}(u)\cdot x(u)\;du,\psi_{s,t}=\frac{1}{\sqrt{s}}\psi \left(\frac{u-t}{s}\right)$$

where $w\left(s,t\right)$ denotes the wavelet coefficients, x(u) is the input time-series signal, and $\psi \left(u\right)$ represents the mother wavelet. The variables *s* and *t* correspond to the scale (dilation) and translation (time shift) parameters, respectively, while $\frac{1}{\sqrt{s}}$ acts as the energy normalization factor [19,27,28].

The Morlet wavelet was selected as the mother wavelet because it provides a good balance between time and frequency localization, making it well-suited for analyzing hemodynamic oscillations in blood flow signals [19]. The Morlet wavelet is defined as [19]

(2) $$\psi (u)=\pi^{-1/4}e^{i\omega_{0}u}e^{-u^{2}/2}$$

where ψ(u) is the wavelet function, $\pi^{-1/4}$ serves as the energy normalization factor, and $\omega_{0}$ denotes the central frequency of the mother wavelet. The terms $e^{i\omega_{0}u}$ and $e^{-u^{2}/2}$ represent the complex sinusoidal oscillation and the Gaussian window envelope, respectively.

Wavelet power was computed as the squared magnitude of the wavelet coefficients [28]:

(3) $$P(s,t)=|w(s,t){|}^{2}$$

where P(s,t) represents the local wavelet power spectrum at scale *s* and time *t*, and w(s,t) denotes the corresponding complex wavelet coefficient.

The CWT was implemented using Multiscale Oscillatory Dynamics Analysis (MODA, version 1.01, Lancaster University, Lancaster, UK) [29]. The frequency range was set from 0.0095 to 0.14 Hz, with the upper limit constrained by the 0.14 Hz low-pass filter applied during data acquisition. Wavelet coefficients within the cone of influence were excluded from subsequent analyses to minimize boundary effects.

Based on the established five-band framework of microvascular blood flow oscillations, three physiological frequency bands were identified within the accessible frequency range [18]: endothelial band (0.0095–0.02 Hz), associated with endothelial cell activity and nitric oxide-mediated vasodilation; neurogenic band (0.02–0.05 Hz), associated with sympathetic nervous system regulation of vascular tone; myogenic band (0.05–0.14 Hz), associated with intrinsic smooth muscle activity of the vessel wall [18]. Band-averaged wavelet power was calculated as the mean squared magnitude of the wavelet coefficients across all time points and frequency bins within each physiological band, following the approach of Hou et al. who computed mean wavelet amplitude across frequency bands to assess blood flow control mechanisms after cupping therapy [19].

#### 2.4.2. Sample Entropy

Sample entropy (SampEn) was computed to quantify the irregularity and complexity of OxyHb and DeoxyHb time series recorded from the areas inside and outside the cupping cup. Sample entropy is a model-independent measure of signal complexity that quantifies the likelihood that similar patterns in a time series will remain similar at the next comparison [21]. Unlike approximate entropy, sample entropy does not count self-matches, making it more consistent and reliable for physiological time series of finite length [21]. The loss of complexity in physiological signals has been associated with aging, disease, and impaired physiological adaptability, while higher complexity is generally considered a marker of a healthy and responsive system [30]. Sample entropy is defined as [21]

(4) $$SampEn(m,r,N)=-ln\left(\frac{C_{m+1}(r)}{C_{m}(r)}\right)$$

where SampEn is the sample entropy value, *m* represents the embedding dimension, *r* denotes the tolerance threshold, and *N* is the total length of the time series. The terms $C_{m}\left(r\right)$ and $C_{m+1}\left(r\right)$ correspond to the probability of finding matching vectors of length *m* and *m* + 1, respectively, within the specified tolerance. Template matching was assessed using the Chebyshev distance, two template vectors are considered to match if the maximum absolute difference between corresponding elements is less than *r* [21]. A higher *SampEn* value indicates greater signal complexity and less predictable hemodynamic behavior, reflecting enhanced physiological adaptability of the microvascular system [30].

Template length was set to *m* = 2 and tolerance was set to *r* = 0.2 × *SD*(*x*), where *SD*(*x*) is the standard deviation of the signal [21]. These values are consistent with the commonly recommended parameters for physiological time series analysis, *m* = 2 and *r* between 0.1 and 0.25 times the standard deviation of the data, as reported in the sample entropy literature [31]. It should be noted that parameter selection can influence entropy estimates, and *r* = 0.2 × *SD* was selected as it falls within the recommended range and has been widely adopted in prior hemodynamic studies [21]. Signal length was *N* = 1200 samples for all subjects and conditions, corresponding to 600 s of post-cupping recording at a sampling rate of 2 Hz.

Sample entropy was selected because it has been validated as a robust complexity descriptor within established methodological frameworks, including the work of Porta et al., where global SampEn tracked progressive changes in cardiac control complexity during physiological challenges. SampEn is also the most widely applied complexity measure in the NIRS and microvascular literature, ensuring direct comparability with prior cupping work, and its model-free formulation makes no assumptions about the underlying signal structure [32].

Sample entropy has been previously applied to characterize the complexity of surface electromyography signals in the context of muscle fatigue and recovery within the same research framework, demonstrating its sensitivity to changes in neuromuscular regulation [33]. This application extends the complexity analysis framework to quantify the dynamic adaptability of muscle hemodynamic responses, providing information that cannot be captured by mean value analyses alone, which, as Lipsitz and Goldberger demonstrated, ignore the dynamic nature of physiological processes [30].

#### 2.4.3. Detrended Fluctuation Analysis

Detrended fluctuation analysis (DFA) was applied to quantify the long-range temporal correlations and fractal scaling properties of OxyHb and DeoxyHb time series recorded from the areas inside and outside the cupping cup. DFA is a method for detecting long-range power-law correlations in non-stationary time series, originally developed for the analysis of DNA nucleotide sequences [34] and subsequently extended to physiological signals such as heart rate variability [22]. It is particularly well suited for signals that exhibit trends that would otherwise confound traditional correlation analyses [22,34]. The DFA scaling exponent α characterizes the fractal properties of the signal and reflects the degree of self-similarity of hemodynamic fluctuations across multiple time scales [22,34].

The DFA procedure followed the method originally described by Peng et al. and consists of four steps. First, the mean-subtracted time series was integrated to produce a cumulative sum profile *Y*(*k*) [22,34]:

(5) $$Y(k)=\sum_{i=1}^{k}\left[x(i)-\bar{x}\right],k=1,2,\ldots ,N$$

where $Y\left(k\right)$ is the integrated cumulative sum profile, *x* represents the *i*-th value of the original time series, $\overset{¯}{x}$ denotes the global mean of the signal, and *N* is the total number of data points. Second, the integrated signal was divided into non-overlapping windows of size *n*, a linear trend was estimated within each window using ordinary least squares regression and subtracted, and the root mean square fluctuation *F*(*n*) was computed across all windows [22,34]:

(6) $$F(n)=\sqrt{\frac{1}{N}\sum_{k=1}^{N}{\left[Y(k)-Y_{n}(k)\right]}^{2}}$$

where *F*(*n*) is the fluctuation function at window scale *n*, *Y*(*k*) represents the integrated cumulative sum profile, $Y_{n}\left(k\right)$ is the local trend derived from a least-squares fit within each window, and *N* denotes the total number of data points. Third, steps one and two were repeated for *log*-spaced window sizes *n* ranging from *n_min_* = 10 samples to *n_max_* = *N*/4 = 300 samples, following standard recommendations [22]. Fourth, the DFA scaling exponent α was estimated as the slope of the linear regression of log *F*(*n*) on log *n* [22,34]:

(7) logF(n)=α⋅log(n)+const

where *F*(*n*) represents the fluctuation function, *n* is the window box size, and const is the regression intercept. The slope, denoted by the scaling exponent α, characterizes the long-range temporal correlations and fractal properties of the hemodynamic time series.

If the signal has long-range power-law correlations, *F*(*n*) scales as a power law with n, and *α* is interpreted as the fractal scaling exponent [22,34].

The scaling exponent *α* is interpreted as follows [22,35]: *α* = 0.5 indicates uncorrelated white noise with no memory; 0.5 < *α* < 1.0 indicates stationary long-range correlations; *α* = 1.0 indicates 1/*f* pink noise, characteristic of healthy physiological regulation; and *α* > 1.0 indicates non-stationary signals with strong persistent long-range correlations, with *α* = 1.5 corresponding to Brownian motion [35]. Healthy physiological systems typically exhibit a scaling exponent near *α* = 1.0, reflecting long-range correlated dynamics [35]. Higher exponents approaching 1.5 have been observed with aging and certain disease states, indicating altered fractal organization [35]. Deviations from scale-free dynamics have been associated with impaired physiological function and reduced adaptability [35]. Accordingly, within the range typically observed for non-stationary physiological signals (1.0 < α < 1.5), lower α values reflect dynamics closer to the healthy 1/*f* reference and are interpreted as indicating more physiologically organized long-range correlated regulation, whereas higher α values reflect a shift toward Brownian-like behavior with reduced regulatory complexity [23].

The application of DFA to physiological signals has been demonstrated in cardiovascular [22], neuromuscular [36], and brain hemodynamic contexts using NIRS [37]. The present study extends this framework to NIRS OxyHb and DeoxyHb signals recorded during cupping therapy, quantifying the long-range temporal organization of muscle hemodynamic responses to mechanical pressure and providing complementary information to the frequency-domain analyses described in previous sections.

### 2.5. Statistical Analysis

A two-way repeated measures analysis of variance (ANOVA) was used to examine the main effects and interaction effects of Pressure (−225 mmHg vs. −300 mmHg) and Duration (5 min vs. 10 min) on each dependent variable, including band-averaged wavelet power, sample entropy, and DFA scaling exponent, measured from the inside-cup region. When a significant main effect or interaction was identified, Bonferroni-corrected pairwise comparisons were performed to determine specific differences between factor levels. For significant main effects of Pressure or Duration, paired *t*-tests were conducted between the corresponding factor levels collapsed across other factors. For significant Pressure × Duration interactions, pairwise comparisons were performed among all four cupping conditions (A: −225 mmHg/5 min, B: −225 mmHg/10 min, C: −300 mmHg/5 min, D: −300 mmHg/10 min). The Bonferroni correction was applied to adjust for multiple comparisons within each family of tests.

Prior to ANOVA, the assumption of sphericity was tested using Mauchly’s test, which also served to verify the assumption of normal distribution of the data, consistent with the analytical approach used in previous studies from our group [25,26]. When the sphericity assumption was violated, the Greenhouse–Geisser corrected *p*-values were reported. The dependent variables for the two-way ANOVA were organized as follows: for wavelet spectral power, the dependent variables were the band-averaged power values in the endothelial (0.0095–0.02 Hz), neurogenic (0.02–0.05 Hz), and myogenic (0.05–0.14 Hz) frequency bands, computed separately for OxyHb and DeoxyHb signals from the inside-cup channels. For sample entropy and DFA, the dependent variables were the SampEn and α values computed separately for OxyHb and DeoxyHb signals from the inside-cup channels. The significance level was set at *p* < 0.05 for all statistical tests. Effect sizes are reported as partial eta squared (η^2^p), where values of 0.01, 0.06, and 0.14 are considered small, medium, and large effects, respectively [38]. All statistical analyses were performed in MATLAB R2025b (The MathWorks Inc., Natick, MA, USA) using the fitrm and ranova functions from the Statistics and Machine Learning Toolbox [39].

## 3. Results

**Figure 1:**
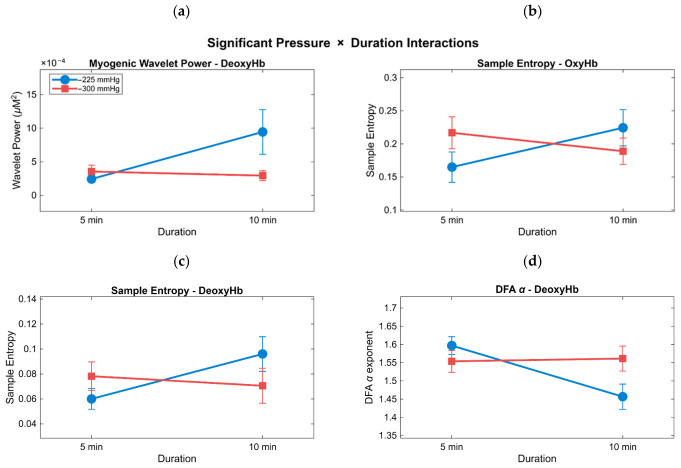
Significant Pressure × Duration interactions on hemodynamic measures within the cupping cup. (**a**) Myogenic-band wavelet power of DeoxyHb (0.05−0.14 Hz; *F* = 4.636, *p* = 0.046, η^2^p = 0.214); (**b**) Sample entropy of OxyHb (*F* = 5.704, *p* = 0.029, η^2^p = 0.251); (**c**) Sample entropy of DeoxyHb (*F* = 6.600, *p* = 0.020, η^2^p = 0.280); (**d**) DFA scaling exponent α of DeoxyHb (*F* = 7.775, *p* = 0.013, η^2^p = 0.314). Data are presented as mean ± standard error of the mean (SEM; *N* = 18). Blue circles indicate the −225 mmHg condition and red squares indicate the −300 mmHg condition.

Figure 1 summarizes the statistically significant Pressure × Duration interactions identified across measures: myogenic wavelet power in DeoxyHb (Figure 1a), sample entropy in OxyHb and DeoxyHb (Figure 1b,c), and DFA scaling exponent α in DeoxyHb (Figure 1d), which also showed a significant Duration main effect. Detailed statistical results are reported in the following subsections.

### 3.1. Wavelet Spectral Power: OxyHb

**Figure 2:**
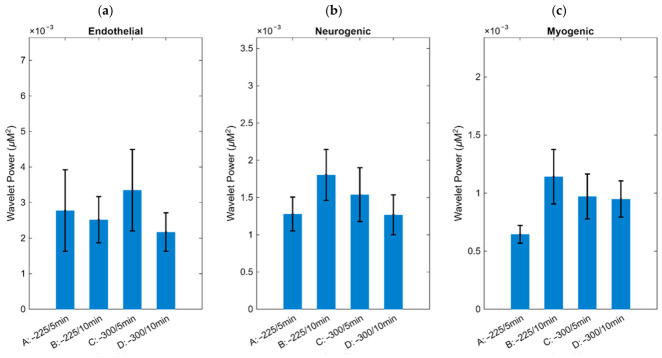
Band-averaged OxyHb wavelet power across four cupping conditions (A: −225 mmHg/5 min, B: −225 mmHg/10 min, C: −300 mmHg/5 min, D: −300 mmHg/10 min) in the (**a**) endothelial (0.0095–0.02 Hz), (**b**) neurogenic (0.02–0.05 Hz), and (**c**) myogenic (0.05–0.14 Hz) frequency bands. No significant main effects of Pressure or Duration, and no significant Pressure × Duration interaction were found for OxyHb wavelet power in any frequency band (all *p* > 0.05). Results are represented as mean ± standard error of the mean.

Figure 2 shows the band-averaged OxyHb wavelet power inside the cupping cup across the four cupping conditions in the three physiological frequency bands. A two-way repeated measures ANOVA with factors Pressure (−225 mmHg vs. −300 mmHg) and Duration (5 min vs. 10 min) revealed no significant main effects or interactions in any frequency band. In the endothelial frequency band (0.0095–0.02 Hz; Figure 2a), no significant effects of Pressure (*F* = 0.024, *p* = 0.879, η^2^p = 0.001), Duration (*F* = 0.749, *p* = 0.399, η^2^p = 0.042), or Pressure × Duration interaction (*F* = 0.509, *p* = 0.485, η^2^p = 0.029) were found, indicating that endothelial-related OxyHb oscillations were not significantly influenced by cupping pressure or duration.

In the neurogenic frequency band (0.02–0.05 Hz; Figure 2b), no significant effects of Pressure (*F* = 0.226, *p* = 0.641, η^2^p = 0.013), Duration (*F* = 0.173, *p* = 0.683, η^2^p = 0.010), or Pressure × Duration interaction (*F* = 2.351, *p* = 0.144, η^2^p = 0.122) were observed, suggesting that neurogenic regulation of OxyHb oscillations was not significantly modulated by the cupping parameters applied in this study.

In the myogenic frequency band (0.05–0.14 Hz; Figure 2c), no significant effects of Pressure (*F* = 0.135, *p* = 0.718, η^2^p = 0.008), Duration (*F* = 2.644, *p* = 0.122, η^2^p = 0.135), or Pressure × Duration interaction (*F* = 2.604, *p* = 0.125, η^2^p = 0.133) were found (Figure 1a). Although a trend toward a duration effect was observed in the myogenic band, it did not reach statistical significance. Overall, OxyHb wavelet power was not significantly modulated by cupping pressure or duration in any of the three physiological frequency bands. The two-way repeated measures ANOVA results for wavelet spectral power are summarized in Table 1.

### 3.2. Wavelet Spectral Power: DeoxyHb

**Figure 3:**
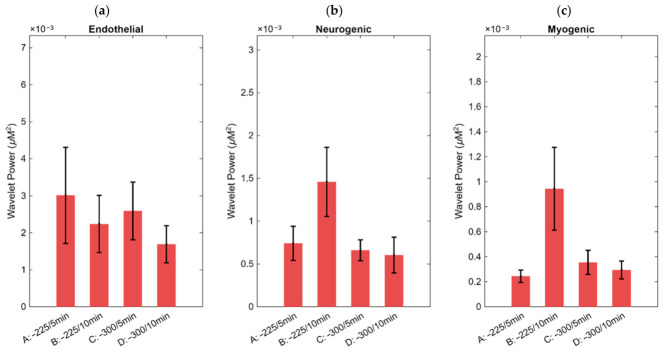
Band-averaged DeoxyHb wavelet power across four cupping conditions (A: −225 mmHg/5 min, B: −225 mmHg/10 min, C: −300 mmHg/5 min, D: −300 mmHg/10 min) in the (**a**) endothelial (0.0095–0.02 Hz), (**b**) neurogenic (0.02–0.05 Hz), and (**c**) myogenic (0.05–0.14 Hz) frequency bands. No significant main effects or interactions were found for DeoxyHb wavelet power in the endothelial band (all *p* > 0.05). In the neurogenic band (**b**), a significant main effect of Pressure was observed (*F* = 5.001, *p* = 0.039, η^2^p = 0.227; however, no individual condition pairs reached significance after Bonferroni correction. In the myogenic band (**c**), a significant Pressure × Duration interaction was found (*F* = 4.636, *p* = 0.046, η^2^p = 0.214); post-hoc pairwise comparisons did not identify specific significant condition pairs after Bonferroni correction. Results are represented as mean ± standard error of the mean.

Figure 3 shows the band-averaged DeoxyHb wavelet power inside the cupping cup across the four cupping conditions in the three physiological frequency bands. In the endothelial frequency band (0.0095–0.02 Hz; Figure 3a), no significant effects of Pressure (*F* = 0.900, *p* = 0.356, η^2^p = 0.050), Duration (*F* = 1.233, *p* = 0.282, η^2^p = 0.068), or Pressure × Duration interaction (*F* = 0.007, *p* = 0.935, η^2^p = 0.000) were found, indicating that endothelial-related DeoxyHb oscillations were not significantly influenced by cupping pressure or duration.

In the neurogenic frequency band (0.02–0.05 Hz; Figure 3b), a significant main effect of Pressure was observed (*F* = 5.001, *p* = 0.039, η^2^p = 0.227), indicating that cupping pressure significantly influenced the neurogenic DeoxyHb oscillations inside the cup. Specifically, condition B (−225 mmHg/10 min) produced notably higher DeoxyHb neurogenic power than conditions C and D (−300 mmHg), suggesting that lower cupping pressure was associated with greater neurogenic vascular regulation. However, no individual condition pairs reached significance after Bonferroni correction (all *p* > 0.05), indicating that the pressure effect was distributed broadly rather than driven by a single dominant pair. No significant effects of Duration (*F* = 2.426, *p* = 0.138, η^2^p = 0.125) or Pressure × Duration interaction (*F* = 3.079, *p* = 0.097, η^2^p = 0.153) were observed, although the interaction showed a trend approaching significance.

In the myogenic frequency band (0.05–0.14 Hz; Figure 3c), a significant Pressure × Duration interaction was found (*F* = 4.636, *p* = 0.046, η^2^p = 0.214), indicating that the combined effects of cupping pressure and duration differentially modulated myogenic DeoxyHb oscillations inside the cup. Condition B (−225 mmHg/10 min) produced the highest myogenic DeoxyHb power among all conditions (mean = 0.00094 μM^2^), while condition A (−225 mmHg/5 min) produced the lowest (mean = 0.00024 μM^2^), suggesting that the duration effect was most pronounced at lower pressure. However, no individual condition pairs reached significance after Bonferroni correction (all *p* > 0.05). No significant main effects of Pressure (*F* = 2.213, *p* = 0.155, η^2^p = 0.115) or Duration (*F* = 3.712, *p* = 0.071, η^2^p = 0.179) were found, although Duration showed a trend approaching significance. The two-way repeated measures ANOVA results for wavelet spectral power are summarized in Table 1.

### 3.3. Sample Entropy

**Figure 4:**
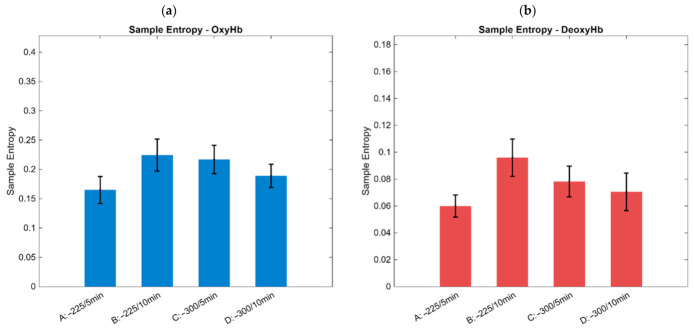
Sample entropy of (**a**) OxyHb and (**b**) DeoxyHb signals across four cupping conditions (A: −225 mmHg/5 min, B: −225 mmHg/10 min, C: −300 mmHg/5 min, D: −300 mmHg/10 min). For OxyHb (**a**), a significant Pressure × Duration interaction was found (*F* = 5.704, *p* = 0.029, η^2^p = 0.251), no individual condition pairs reached significance after Bonferroni correction. No significant main effects of Pressure (*F* = 0.114, *p* = 0.740) or Duration (*F* = 0.384, *p* = 0.544) were observed for OxyHb. For DeoxyHb (**b**), a significant Pressure × Duration interaction was also found (*F* = 6.600, *p* = 0.020, η^2^p = 0.280), no individual condition pairs reached significance after Bonferroni correction. No significant main effects of Pressure (*F* = 0.083, *p* = 0.777) or Duration (*F* = 1.501, *p* = 0.237) were observed for DeoxyHb. Results are represented as mean ± standard error of the mean.

Figure 4 shows the sample entropy of OxyHb and DeoxyHb signals inside the cupping cup across the four cupping conditions. For OxyHb sample entropy (Figure 4a), a significant Pressure × Duration interaction was found (*F* = 5.704, *p* = 0.029, η^2^p = 0.251) (Figure 1b), indicating that the combined effects of cupping pressure and duration differentially modulated the complexity of OxyHb hemodynamic signals inside the cup. Condition B (−225 mmHg/10 min) produced the highest OxyHb sample entropy (mean = 0.224), followed by condition C (−300 mmHg/5 min; mean = 0.217), condition D (−300 mmHg/10 min; mean = 0.189), and condition A (−225 mmHg/5 min; mean = 0.165), suggesting that longer duration at low pressure and shorter duration at high pressure were both associated with greater signal complexity. However, no individual condition pairs reached significance after Bonferroni correction (all *p* > 0.05), indicating that the interaction effect was broadly distributed rather than driven by a single dominant condition pair. No significant main effects of Pressure (*F* = 0.114, *p* = 0.740, η^2^p = 0.007) or Duration (*F* = 0.384, *p* = 0.544, η^2^p = 0.022) were observed for OxyHb sample entropy.

For DeoxyHb sample entropy (Figure 4b), a significant Pressure × Duration interaction was also found (*F* = 6.600, *p* = 0.020, η^2^p = 0.280) (Figure 1c), indicating that the combined influence of cupping pressure and duration significantly modulated the complexity of DeoxyHb signals. Condition B (−225 mmHg/10 min) produced the highest DeoxyHb sample entropy (mean = 0.096), followed by condition C (−300 mmHg/5 min; mean = 0.078), condition D (−300 mmHg/10 min; mean = 0.071), and condition A (−225 mmHg/5 min; mean = 0.060), showing a pattern consistent with the OxyHb findings. Similarly, no individual condition pairs reached significance after Bonferroni correction (all *p* > 0.05). No significant main effects of Pressure (*F* = 0.083, *p* = 0.777, η^2^p = 0.005) or Duration (*F* = 1.501, *p* = 0.237, η^2^p = 0.081) were found for DeoxyHb sample entropy.

The consistent Pressure × Duration interaction observed in both OxyHb and DeoxyHb sample entropy suggests that the dynamic complexity of muscle hemodynamic signals is jointly determined by the combination of cupping pressure and duration, rather than by either factor independently. The two-way repeated measures ANOVA results for sample entropy are summarized in Table 2.

### 3.4. Detrended Fluctuation Analysis

**Figure 5:**
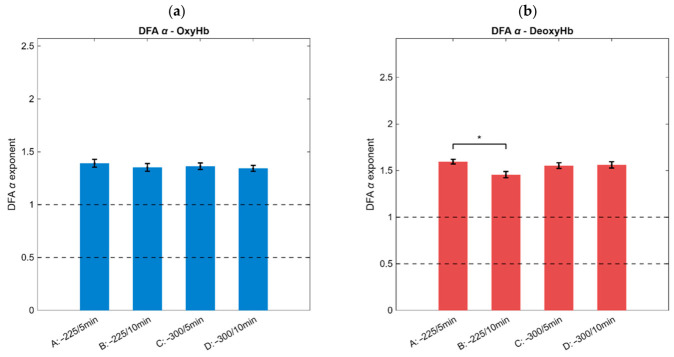
DFA scaling exponent α of (**a**) OxyHb and (**b**) DeoxyHb signals across four cupping conditions (A: −225 mmHg/5 min, B: −225 mmHg/10 min, C: −300 mmHg/5 min, D: −300 mmHg/10 min). Dashed lines indicate reference values of *α* = 0.5 (uncorrelated white noise) and *α* = 1.0 (healthy long-range correlated dynamics). All *α* values ranged between 1.30 and 1.65 across all conditions. For (**a**) OxyHb, no significant main effects of Pressure (*F* = 0.279, *p* = 0.604), Duration (*F* = 0.863, *p* = 0.366), or Pressure × Duration interaction (*F* = 0.152, *p* = 0.701) were found. For (**b**) DeoxyHb, a significant main effect of Duration was observed (*F* = 5.598, *p* = 0.030, η^2^p = 0.248). A significant Pressure × Duration interaction was also found (*F* = 7.775, *p* = 0.013, η^2^p = 0.314); Bonferroni-corrected pairwise comparisons revealed that condition A (−225 mmHg/5 min) had a significantly higher DeoxyHb *α* than condition B (−225 mmHg/10 min; *p* = 0.011). Results are represented as mean ± standard error of the mean. * *p* < 0.05.

Figure 5 presents the DFA scaling exponent α for OxyHb and DeoxyHb signals inside the cupping cup across the four cupping conditions. All α values ranged between 1.30 and 1.65 across all subjects and conditions, well above the reference value of α = 0.5 (uncorrelated white noise) and above α = 1.0 (healthy long-range correlated pink noise dynamics), indicating strong persistent long-range temporal correlations in all hemodynamic signals consistent with healthy physiological regulation. Values in this range are characteristic of non-stationary signals with strong fractal scaling properties, approaching but not reaching α = 1.5 (Brownian motion).

For OxyHb (Figure 5a), no significant main effects of Pressure (*F* = 0.279, *p* = 0.604, η^2^p = 0.016), Duration (*F* = 0.863, *p* = 0.366, η^2^p = 0.048), or Pressure × Duration interaction (*F* = 0.152, *p* = 0.701, η^2^p = 0.009) were found. OxyHb α values ranged from 1.34 to 1.39 across all conditions, remaining stable and consistently above α = 1.0, indicating that the fractal scaling properties of OxyHb signals were not significantly modulated by cupping pressure or duration.

For DeoxyHb (Figure 5b), a significant main effect of Duration was observed (*F* = 5.598, *p* = 0.030, η^2^p = 0.248), indicating that cupping duration significantly influenced the fractal scaling properties of DeoxyHb signals. Shorter cupping duration (5 min) was associated with higher α values than longer duration (10 min), suggesting that prolonged cupping reduced the strength of long-range temporal correlations in DeoxyHb signals, shifting α values toward α = 1.0 (pink noise) from the higher range approaching α = 1.5 (Brownian motion). A significant Pressure × Duration interaction was also found (*F* = 7.775, *p* = 0.013, η^2^p = 0.314) (Figure 1d), indicating that the combined effects of pressure and duration jointly determined the fractal scaling of DeoxyHb signals. Bonferroni-corrected pairwise comparisons revealed that condition A (−225 mmHg/5 min; mean α = 1.597) had significantly higher DeoxyHb α than condition B (−225 mmHg/10 min; mean α = 1.457; *p* = 0.011), indicating that increasing duration from 5 to 10 min at low pressure (−225 mmHg) substantially reduced the long-range temporal correlations of DeoxyHb signals. No other condition pairs reached significance after Bonferroni correction (all *p* > 0.05), indicating that the duration effect on DeoxyHb fractal scaling was specifically present at low pressure (−225 mmHg) but not at high pressure (−300 mmHg). No significant main effect of Pressure was found (*F* = 0.702, *p* = 0.414, η^2^p = 0.040). The two-way repeated measures ANOVA results for DFA scaling exponent are summarized in Table 3.

## 4. Discussion

This study investigated the effects of cupping pressure and duration on the time-frequency structure, complexity, and fractal scaling of OxyHb and DeoxyHb hemodynamic responses in the biceps muscle using NIRS. To the best of our knowledge, this is the first study to apply time-frequency analysis and complexity analysis, specifically wavelet transform [40], sample entropy [21], and detrended fluctuation analysis [22], to NIRS hemodynamic signals to characterize the mechanisms of action of cupping therapy in terms of microvascular frequency-specific oscillations and signal dynamics.

The results demonstrated that the combined effects of pressure and duration, rather than either factor independently, were the primary determinants of the dynamic hemodynamic response to cupping therapy, with significant Pressure × Duration interactions observed in DeoxyHb myogenic wavelet power, OxyHb and DeoxyHb sample entropy, and DeoxyHb DFA scaling exponent. In addition, cupping pressure selectively modulated neurogenic DeoxyHb oscillatory power, and cupping duration significantly altered the fractal scaling properties of DeoxyHb signals. These findings complement and extend the time-domain NIRS analyses previously reported by our group [25,26] by revealing that the influence of cupping parameters extends beyond changes in mean hemoglobin values to encompass the dynamic structure, oscillatory regulation, and long-range temporal organization of muscle hemodynamic signals.

The significant Pressure × Duration interactions observed across multiple hemodynamic measures suggest that the response to cupping therapy is not simply additive, rather, pressure and duration interact in a complex manner to jointly shape both the oscillatory structure and the dynamic complexity of the hemodynamic signals. Specifically, condition B (−225 mmHg/10 min) consistently produced the highest sample entropy values for both OxyHb and DeoxyHb, indicating greater signal complexity and physiological adaptability at lower pressure combined with longer duration [21,35]. By contrast, condition A (−225 mmHg/5 min) produced the highest DeoxyHb DFA scaling exponent, suggesting that shorter duration at the same pressure is associated with stronger long-range temporal correlations [22]. This pattern suggests that at lower pressure, longer duration enhances the complexity and adaptability of hemodynamic signals, potentially reflecting greater physiological responsiveness of the microvascular system to sustained but moderate mechanical stimulation. At higher pressure (−300 mmHg), the duration effect on DeoxyHb fractal scaling was absent, suggesting that high mechanical pressure may override or saturate the duration-dependent vascular response, limiting the capacity of the microvascular system to further adapt with increasing duration. This finding is consistent with the Pressure × Duration interactions previously reported in time-domain analyses of cupping therapy from our group, where significant interactions were observed for DeoxyHb mean values [25,26]. The Pressure × Duration interaction in myogenic DeoxyHb wavelet power specifically implicates vascular smooth muscle activity as a key mechanism [41] through which the combination of mechanical pressure and duration modulates microvascular regulation under the cup. Hou et al. previously demonstrated using laser Doppler flowmetry with wavelet analysis that cupping therapy weakened the regulation of vascular smooth muscles on vasomotion in skin blood flow, with larger cup sizes reinforcing this effect [19]. The present findings extend this observation to muscle hemodynamics measured by NIRS, suggesting that the degree of smooth muscle regulatory modulation is jointly determined by the specific combination of cupping pressure and duration rather than being a uniform response to cupping stimulation.

A significant main effect of Pressure was found for DeoxyHb neurogenic wavelet power, with lower pressure (−225 mmHg) associated with higher neurogenic DeoxyHb oscillatory power than higher pressure (−300 mmHg). This finding suggests that lower cupping pressure preferentially activates sympathetic nervous system regulation of the microvasculature, as reflected in the neurogenic frequency band (0.02–0.05 Hz) [40]. Higher mechanical pressure may partially suppress or override neurogenic vascular tone through direct mechanical deformation of the vessel wall that reduces the relative contribution of sympathetically mediated oscillations. Previous studies from our group using time-domain NIRS analysis reported that higher cupping pressure was associated with greater overall changes in blood volume and oxygenation [25], but did not distinguish between the contributions of different vascular regulatory mechanisms. The present frequency-domain analysis provides a more mechanistically specific characterization by demonstrating that the pressure effect is selectively expressed in the neurogenic rather than the endothelial or myogenic frequency bands for DeoxyHb. The absence of a significant pressure effect on OxyHb neurogenic power, in contrast to the significant effect observed for DeoxyHb, suggests differential sensitivity of the two hemoglobin signals to cupping pressure in the neurogenic frequency band. This differential response is consistent with previous computational analyses demonstrating that OxyHb and DeoxyHb NIRS signals exhibit different sensitivities to blood volume changes in skeletal muscle under varying oxygen delivery conditions [42]. The specific mechanisms underlying this differential neurogenic response to cupping pressure warrant further investigation.

A significant main effect of the duration factor was observed for DeoxyHb DFA scaling exponent, with shorter cupping duration (5 min) associated with higher α values than longer duration (10 min). Since all α values ranged above the healthy reference of α = 1.0 (pink noise) and approached α = 1.5 (Brownian motion) [22], the duration-induced reduction in α represents a shift toward healthier long-range correlated dynamics closer to optimal physiological regulation [22,35]. This may show that longer cupping duration promotes a more physiologically organized fractal structure in DeoxyHb dynamics, potentially reflecting enhanced vascular regulatory efficiency after sustained mechanical stimulation [43].

Notably, the duration effect was specific to low pressure, the significant reduction in α with longer duration was only present at −225 mmHg and was absent at −300 mmHg. This pressure-specific pattern suggests that lower mechanical pressure allows the vascular system sufficient regulatory freedom to respond to sustained stimulation over time, while higher pressure may mechanically constrain the vessel wall in a way that limits duration-dependent adaptation. These findings suggest that an appropriate combination of pressure and duration may be needed to elicit favorable effects of cupping therapy on microvascular dynamics, with 10-min duration at lower pressure appearing particularly effective for promoting favorable DeoxyHb fractal dynamics. Previous studies reported duration-dependent effects of cupping on mean blood flow and oxygenation [25,26] but the present results are the first to demonstrate that duration specifically alters the fractal scaling properties of DeoxyHb signals, providing a new mechanistic perspective on the temporal dynamics of cupping therapy.

The findings of this study have several potential clinical implications for the application of cupping therapy in rehabilitation and sports medicine. Previous work by Li et al. demonstrated that higher cupping pressure (−300 mmHg) was more effective at increasing oxyhemoglobin concentration, indicating greater overall blood volume and perfusion under higher mechanical pressure [25]. The present observation that lower cupping pressure (−225 mmHg) at longer duration (10 min) produced greater hemodynamic complexity and lower fractal scaling exponents might suggest that this combination may be more effective for promoting microvascular responsiveness and adaptability than higher pressure protocols. These may enhance oxygen delivery, while lower pressure with longer duration may optimize the temporal structure and regulatory efficiency of microvascular dynamics. Clinicians may therefore consider using lower negative pressure (e.g., −225 mmHg rather than pressures exceeding −300 mmHg) when the therapeutic goal is to enhance the dynamic regulation of muscle blood flow rather than simply increase mean perfusion. Specific physiological benefits of enhanced microvascular responsiveness include more efficient local blood flow distribution, improved oxygen extraction, faster metabolic waste removal, and greater capacity to match perfusion to metabolic demand [42,44].

The frequency-specific nature of the pressure effect, selectively expressed in the neurogenic band for DeoxyHb, suggests that cupping therapy may engage sympathetically mediated vascular regulation as a key mechanism of action, a finding with potential relevance for the following clinical populations: involving sympathetic dysregulation of microvascular tone, such as chronic muscle pain, pressure ulcer prevention, and post-exercise recovery [19,26]. Chronic pain syndromes with sympathetic overactivity show sustained vasoconstriction and impaired tissue oxygenation [45]. Pressure ulcer risk populations, including individuals with spinal cord injury and older adults, show impaired neurogenic microvascular reactivity that reduces protective hyperemia following mechanical loading [46]. In post-exercise recovery, restoration of microvascular regulatory complexity is associated with faster return to baseline [36].

The present findings can be interpreted within the TCM framework of cupping therapy [1,3]. Unlike mean hemoglobin values, sample entropy and DFA capture blood flow’s dynamic regulatory structure, adaptability and long-range organization, which corresponds more directly to the TCM concept of harmonious, actively regulated circulation. The selective modulation of neurogenic DeoxyHb power by pressure is consistent with unblocking meridians; the Pressure × Duration interaction in myogenic power and sample entropy with promoting blood circulation and removing blood stasis; and the duration-dependent DFA scaling with restoring dynamically balanced microvascular regulation [30,35]. These mappings are proposed as theoretically grounded interpretations based on established physiological mechanisms, not as direct empirical tests of TCM theory.

All three analyses (wavelet transform, sample entropy, and DFA) were applied to the same *N* = 1200-sample post-cupping segment (600 s at 2 Hz). At this length, sample entropy with *m* = 2 and *r* = 0.2 × *SD* is well within the reliable range [21], and DFA window sizes from *n_min_* = 10 to *n_max_* = *N*/4 = 300 samples yield approximately 1.5 decades of scaling, meeting the standard minimum for stable scaling exponent estimation [22,47]. The upper bound of *n_max_* = 150 s does, however, limit characterization of very slow long-range correlations; DFA estimates here therefore reflect scaling behavior over short-to-intermediate time scales, and future studies with extended recordings would allow assessment of slower scales.

Future work could extend this framework via (a) local sample entropy [48] to resolve pattern-specific dynamics; (b) k-nearest-neighbor conditional entropy estimators [32] when embedding dimension varies; and (c) bivariate coupling analyses between OxyHb and DeoxyHb to capture their directional interaction as a readout of integrated microvascular regulation.

Several limitations of this study should be acknowledged. First, only two negative pressure levels (−225 mmHg and −300 mmHg) and two durations (5 min and 10 min) were tested, and it is unclear whether different combinations of pressure and duration outside this range, or intermediate values within it, would produce similar hemodynamic effects. Future studies examining a wider range of pressure–duration combinations are needed to identify the full dose–response relationship and to determine clinically optimal cupping parameters. Second, the sample size of 18 healthy young adults limits the generalizability of the findings to clinical populations, including individuals with musculoskeletal disorders, chronic pain, or impaired microvascular function. Future research should reproduce our study protocols using a diverse population with a larger sample size. Last, the influences of the research location of experiments and the lifestyle, diet and emotion of the participants could affect the outcomes of this study. In this study, the use of crossover study design was implemented to minimize these effects on oxyhemoglobin and deoxyhemoglobin responses. Future research should examine these factors on hemodynamic response to cupping therapy.

## 5. Conclusions

This study is the first to apply wavelet transform, sample entropy, and detrended fluctuation analysis to NIRS signals to characterize the effects of cupping therapy on muscle microvascular dynamics. The results demonstrate that the hemodynamic response to cupping is jointly determined by the combination of pressure and duration, with significant Pressure × Duration interactions observed in DeoxyHb myogenic wavelet power, OxyHb and DeoxyHb sample entropy, and DeoxyHb DFA scaling exponent. Additionally, lower cupping pressure selectively enhanced neurogenic DeoxyHb oscillatory power, and longer duration promoted more physiologically organized fractal scaling in DeoxyHb signals. These findings extend prior time-domain analyses by revealing that cupping therapy modulates not only mean hemoglobin values but also the dynamic complexity and oscillatory regulation of muscle hemodynamic signals. Clinically, lower pressure with longer duration appears more effective for optimizing microvascular regulatory dynamics, while higher pressure may better serve protocols aimed at increasing mean tissue perfusion. Future studies should examine a wider range of cupping parameters and include clinical populations to validate and extend these findings.

## Figures and Tables

**Table 1:** Two-way ANOVA results: wavelet spectral power.

	*F*	*p*	η^2^p	*F*	*p*	η^2^p	*F*	*p*	η^2^p
	Pressure × Duration			Pressure			Duration		
**Endothelial band (0.0095–0.02 Hz)**									
**OxyHb**	0.509	0.485	0.029	0.024	0.879	0.001	0.749	0.399	0.042
**DeoxyHb**	0.007	0.935	0.000	0.900	0.356	0.050	1.233	0.282	0.068
**Neurogenic band (0.02–0.05 Hz)**									
**OxyHb**	2.351	0.144	0.122	0.226	0.641	0.013	0.173	0.683	0.010
**DeoxyHb**	3.079	0.097	0.153	5.001	0.039 *	0.227	2.426	0.138	0.125
**Myogenic band (0.05–0.14 Hz)**									
**OxyHb**	2.604	0.125	0.133	0.135	0.718	0.008	2.644	0.122	0.135
**DeoxyHb**	4.636	0.046 *	0.214	2.213	0.155	0.115	3.712	0.071	0.179

* *p* < 0.05. Effect size reported as partial η^2^p. *F* values are Greenhouse–Geisser corrected where sphericity was violated. Bold values denote statistical significance.

**Table 2:** Two-way ANOVA results: sample entropy.

	*F*	*p*	η^2^p	*F*	*p*	η^2^p	*F*	*p*	η^2^p
	Pressure × Duration			Pressure			Duration		
**OxyHb**	5.704	0.029 *	0.251	0.114	0.740	0.007	0.384	0.544	0.022
**DeoxyHb**	6.600	0.020 *	0.280	0.083	0.777	0.005	1.501	0.237	0.081

* *p* < 0.05. Effect size reported as partial η^2^p. *F* values are Greenhouse–Geisser corrected where sphericity was violated. Bold values denote statistical significance.

**Table 3:** Two-way ANOVA results: DFA scaling exponent α.

	*F*	*p*	η^2^p	*F*	*p*	η^2^p	*F*	*p*	η^2^p
	Pressure × Duration			Pressure			Duration		
**OxyHb**	0.152	0.701	0.009	0.279	0.604	0.016	0.863	0.366	0.048
**DeoxyHb**	7.775	0.013 *	0.314	0.702	0.414	0.040	5.598	0.030 *	0.248

* *p* < 0.05. Effect size reported as partial η^2^p. *F* values are Greenhouse–Geisser corrected where sphericity was violated. Bold values denote statistical significance.

## Data Availability

The data presented in this study are available on request from the corresponding author due to privacy and ethical restrictions.
